# Fast Forward. People development in Africa

**DOI:** 10.1186/2052-3211-7-S1-O20

**Published:** 2014-12-17

**Authors:** Abré van Buuren, Colette Wessels

**Affiliations:** 1 Imperial Health Sciences, Johannesburg, South Africa; 2 Imperial Logistics, Johannesburg, South Africa

## Background

Supply chain management training remains the most critical discipline in building capacity and ensuring competitiveness and sustainability of Africa in the global context. Imperial Logistics continues to play a significant role through the development of our Fast Forward initiative. While ensuring effective operations and best practice, the company further develops sustainable platforms and solutions to expand our reach and address future skills for continued industry growth and innovation.

## Method

The Imperial Logistics Academy aims to provide integrated, customised training and skills development programmes to Imperial Logistics employees. Imperial Logistics initiated an accreditation project with TETA in order to position the Imperial Logistics Academy as an Institute for Sector Occupational Excellence. With a vision to expand Fast Forward into Africa through focusing consistently on training and skills development for African countries, Imperial Logistics works through Imperial Health Sciences Supply Chain Academy as its primary implementation partner.

## Results

The Fast Forward initiative contributes by providing: full learnership across occupational categories ranging from National Quality Framework education levels 1 – 7, specific training aligned to standards, SOPs and business requirements, a legal framework through institutional accreditation and registration processes, a comprehensive quality assurance function and the use of its quality management system, alignment with human resources strategies, reduced duplication of efforts through the use of existing material and programmes while pooling pockets of excellence, improved skills and operational competencies, career development, personal empowerment and job satisfaction, and improved supply chain performance.

## Discussion

Imperial Logistics remains committed to consistent investment that fast tracks capability development in the African supply chain management industry. The establishment of the Imperial Logistics Academy in combination with ISOE accreditation activities, Ikaheng acquisition, and Imperial Health Science Supply Chain Academy activities in Africa has taken the Imperial Logistics’ Fast Forward initiative to a new level.

## Lessons learned

Through continuous development and building further credibility as a learning organisation, Imperial Logistics further distinguishes itself as an “Employer of Choice” and an industry leader in logistics and supply chain management. Utilising internal small and medium enterprises builds capacity while promoting career development and succession planning, strengthening organisational capability. In addition, the increasing duplication of the framework and utilisation of existing infrastructure improves return on investment and allows for continuous improvement.

